# Clinical Outcomes of Hyperbaric Oxygen Therapy for Diabetic Foot Ulcers: A Systematic Review

**DOI:** 10.7759/cureus.78655

**Published:** 2025-02-06

**Authors:** Ujwala Damineni, Shravani Divity, Sri Ram Charan Gundapaneni, Rithwik Goud Burri, Tejaswi Vadde

**Affiliations:** 1 General Medicine, Maheshwara Medical College and Hospital, Hyderabad, IND; 2 General Medicine, Government Medical College, Mahabubnagar, IND; 3 General Medicine, Rangaraya Medical College, Kakinada, IND; 4 General Medicine, SVS Medical College, Mahabubnagar, IND

**Keywords:** diabetes mellitus, diabetic foot ulcers, hyperbaric oxygen therapy, ischemia, standard care

## Abstract

Diabetic foot ulcers (DFUs) are severe complications of diabetes mellitus that often lead to nontraumatic limb amputation. This systematic review aimed to assess the primary clinical evidence supporting hyperbaric oxygen therapy (HBOT) in the management of DFUs. A literature search was conducted using PubMed, Scopus, and Web of Science from June to August 2024, and six studies with a total of 391 patients were included in the final analysis, after applying relevant inclusion and exclusion criteria.

The majority of the studies indicated reduced major amputation rates, improved ulcer healing rates, and decreased ulcer size and depth with HBOT compared to standard care (SC). To assess the risk of bias, this review used the Cochrane Risk of Bias (RoB 2.0) tool for randomized controlled trials (RCTs) and the Risk of Bias in Non-Randomized Studies of Interventions (ROBINS-I) tool for observational studies. This evaluation uncovered variations in methodological rigor across the included studies in the review. Most studies indicate that HBOT leads to lower rates of major amputations, better ulcer healing, and reduced ulcer dimensions than SC. However, one study found no significant differences in amputation rates or long-term wound healing between groups. Selection bias from inconsistent patient allocation is a common limitation in observational studies, potentially distorting comparisons. Performance bias, particularly insufficient blinding, could have influenced treatment adherence and wound care practices, thereby affecting outcome evaluations. These biases, coupled with differences in SC practices, make it challenging to interpret the true efficacy of HBOT and restrict its clinical applicability. While most studies showed a low risk of bias in certain areas, moderate-to-high bias in key aspects necessitated careful interpretation.

Future high-quality RCTs with stringent blinding, standardized protocols, and defined patient selection criteria are crucial to confirm the effectiveness of HBOT, improve guidelines, and establish its long-term viability. Although this review suggests that HBOT may be valuable for DFUs, additional rigorous research is needed to reduce bias, enhance methodological consistency, and improve the reliability of the findings for clinical implementation.

## Introduction and background

Diabetic foot ulcers (DFUs) are prevalent, severe complications of diabetes mellitus that are caused by factors such as pressure, shear stress, neuropathy, and peripheral arterial disease [1,2]. Up to 34% of individuals with type 2 diabetes develop ulcers, making DFUs the leading cause of nontraumatic limb amputation [2]. Therefore, preventive management strategies, including patient education and early treatment, are crucial [2,3].

Behavioral risk factors, such as smoking, physical inactivity, and inadequate footwear, have been identified, while genetic polymorphisms and elevated plasma thrombin-activatable fibrinolysis inhibitor levels are not directly linked to DFU formation [4,5]. Genetic elements influence wound healing and DFU progression. Genes related to inflammation, collagen synthesis, and tissue regeneration regulate the interplay between genetics and wound repair. Genetic differences can alter inflammatory reactions, collagen formation, and tissue renewal, vital for effective healing. Inflammation and reactive oxygen species (ROS) hinder DFUs' healing. Innovative approaches, like hydrogels with immune-modulating properties, target genetic and molecular mechanisms to mitigate these effects. Hydrogels that lower glucose levels and neutralize ROS improve healing by disrupting the ROS-inflammation cycle and modulating macrophage activity, which is essential for the genetic regulation of healing [6]. DFUs require comprehensive management, involving biomechanical stress monitoring, glycemic control, and behavioral risks [1,4].

DFUs often result from excessive stress on the sole tissues of the foot due to high pressure or intense physical activity, particularly in patients with diabetes and peripheral neuropathy, which reduces foot sensation [7]. Repeated pressure and shear forces on the plantar area during weight-bearing activities cause plantar tissue stress. Peripheral neuropathy decreases protective sensitivity and increases plantar tissue stress through adverse changes in the gait, soft tissues, and foot structure. Untreated stress damages the underlying tissue, causing DFUs [8].

Current treatments of DFUs include debridement, negative-pressure wound therapy, and various pharmacological agents [9,10]. Gene expression analysis identified patterns associated with delayed wound healing, infection susceptibility, and inflammatory responses. Research has identified that immune-related genes that are activated in DFUs, such as CXCL11, IFI44, and IFI44L, indicate ongoing inflammation and delayed healing compared to faster-healing oral ulcers [11]. Stem cell therapy offers regenerative potential through enhanced angiogenesis, fibroblast proliferation, and extracellular matrix remodeling. Stem cells show promise in improving vascularization and tissue regeneration, which are crucial for chronic wound healing [12]. Unlike gene expression analysis, stem cell therapy actively promotes wound healing, making it a potential adjunct treatment for DFUs [13,14]. Effective DFU management requires an interdisciplinary strategy tailored to the needs of individual patients. Continued research is critical for improving healing outcomes and addressing the challenges posed by multidrug-resistant bacteria and biofilms [9,10]. Emerging therapies show promise, but further investigation is required to confirm their efficacy and integration into clinical practice [13].

Injuries of this nature often require prolonged healing times because of widespread infections from various microbial agents compounded by compromised immune systems and high levels of antibiotic resistance [15]. Chronic ulcers cause tissue hypoxia and have impaired healing, highlighting the critical role of effective ulcer management during recovery. Consequently, hyperbaric oxygen therapy (HBOT) has been suggested as a beneficial adjunct to DFU treatment. This therapy involves patients inhaling pure oxygen at two to three times the normal atmospheric pressure in a hyperbaric chamber, thereby increasing arterial and tissue oxygen levels. Researchers recommend HBOT for ulcers unresponsive to standard care (SC), particularly those with Wagner grade ≥ 3 that do not improve after 30 days of conventional treatment [16,17].

Patients with chronic ulcers treated with HBOT show several positive physiological changes, including enhanced angiogenesis, improved collagen synthesis, increased white blood cell activity, and reduced swelling. HBOT improves local tissue oxygenation, mitigates hypoxia, and enhances transcutaneous oxygen pressure levels. The antimicrobial properties of HBOT reduce infection risk, expedite healing, and prevent amputation [18]. These effects occur through enhanced leukocyte function, increased oxygenation of deprived tissues, and altered immune response. Increased oxygen availability boosts the oxidative killing mechanism of neutrophils, enhances phagocytosis, and improves leukocyte elimination [19]. HBOT also reduces tissue swelling and improves blood flow to ischemic areas, making the conditions less favorable for anaerobic bacteria. Despite these benefits, HBOT is not a substitute for conventional antimicrobial treatments like antibiotics or surgical removal of dead tissue. It complements therapy by optimizing the host immune response and enhancing infection management strategies.

Treatment with HBOT for DFUs remains controversial, with some studies mentioning that it is ineffective for chronic ischemic or neuroischemic ulcers, suggesting that resources should be redirected to more promising treatments. Several studies have indicated that HBOT provides no significant advantages over standard treatments in healing DFUs or in reducing the risk of amputation, resulting in insufficient scientific evidence to support healthcare professionals in its therapeutic use [20]. This study aimed to assess the primary clinical evidence supporting the use of HBOT in the management of DFU.

## Review

Materials and methods

A systematic review is a comprehensive study that methodically collects, evaluates, synthesizes, and presents the findings from multiple studies on a specific research question [21]. In this study, we employed the Preferred Reporting Items for Systematic Reviews and Meta-Analyses (PRISMA) methodology using its 27-item checklist [22]. The research question was “What is the main clinical evidence for HBOT in treating DFUs?”

In November 2024, a comprehensive literature search was performed using three databases: PubMed, Scopus, and Web of Science. The search employed MeSH terms, including 'hyperbaric oxygenation, ’ 'diabetes, ’ 'diabetic foot, leg ulcers, and’ ischemia.’ To refine the search results, the Boolean operators 'AND' and 'OR' were used to combine descriptors specific to each database. The search was limited to studies published between January 2016 and May 2024. The selection criteria for this study included full-text articles written in English.

This review examines research on systemic HBOT for DFUs. It included studies on adults diagnosed with DFUs, particularly Wagner grade 3 or higher, or persistent nonhealing ulcers. Systemic HBOT was investigated at pressures of 1.0 atmosphere absolute (ATA) or greater as standalone or complementary therapy, compared with SC, placebo HBOT, or alternative treatments. HBOT treatments typically consisted of 20 to 40 sessions, each lasting 60 to 120 min at pressures of 2.0 to 2.5 ATA. Most treatment plans are scheduled sessions daily, five-six times weekly. Follow-up evaluations were conducted from two weeks to 12 months. SC involves wound cleaning, dressing, infection management, pressure relief, and blood sugar control. Studies have reported primary clinical outcomes such as complete wound healing rates, ulcer dimension reduction, amputation frequency, duration until wound closure, or survival without amputation. Secondary outcomes included infection resolution, transcutaneous oxygen pressure changes, and HBOT-related side effects. This review included randomized controlled trials (RCTs) and observational studies. Only complete peer-reviewed English-language articles published between January 2016 and November 2024 were considered. These criteria were aimed at enhancing the clinical applicability, reliability, and generalizability of the results.

Studies were eliminated after removing duplicates, primarily because of unsuitable research designs. These included case reports, editorials, literature reviews, and nonclinical investigations that lacked primary data on HBOT outcomes in DFUs. Studies without crucial primary outcome data, such as ulcer healing rates, amputation rates, or wound size reduction, were excluded. Studies were discarded owing to insufficient statistical analyses, including unclear methodologies, absence of control groups, or inadequate follow-up periods. Studies evaluating topical HBOT rather than systemic HBOT were excluded because they did not align with the review's focus. Research on nondiabetic ulcers was omitted to preserve the specificity of the study population. These exclusion criteria ensured that only high-quality, methodologically sound studies were included, thus enhancing the reliability and generalizability of the conclusions.

Three independent reviewers selected studies based on the titles and abstracts and documented their choices on a research eligibility form. Any disagreements were resolved by consensus. The references were subsequently transferred to an online EndNote management system. Variables, including identification data (title, authors, journal, publication year, and country), methodology (study type and evidence level), number of participants, number of HBOT sessions, follow-up duration, average participant age, and key outcomes (amputation rate, complete DFU healing rate, healing time in days, and mortality rate) were examined.

Standardized tools, including the Cochrane Risk of Bias (RoB 2.0) tool for RCTs and the Risk of Bias in Non-Randomized Studies of Interventions (ROBINS-I) tool for observational studies, were used to assess the quality and risk of bias in the included studies. The RoB 2.0 tool was used to evaluate domains such as selection, performance, detection, attrition, reporting, and other biases. The ROBINS-I tool was used to assess similar domains, including confounding factors, participant selection, intervention classification, deviations from intended interventions, missing data, and selective outcome reporting. Two reviewers independently assessed the risk of bias, with disagreements resolved through discussion or by involving a third reviewer. Each domain was classified as low-, moderate-, or high-risk, and the overall risk was determined for each study.

Results

**Figure 1:**
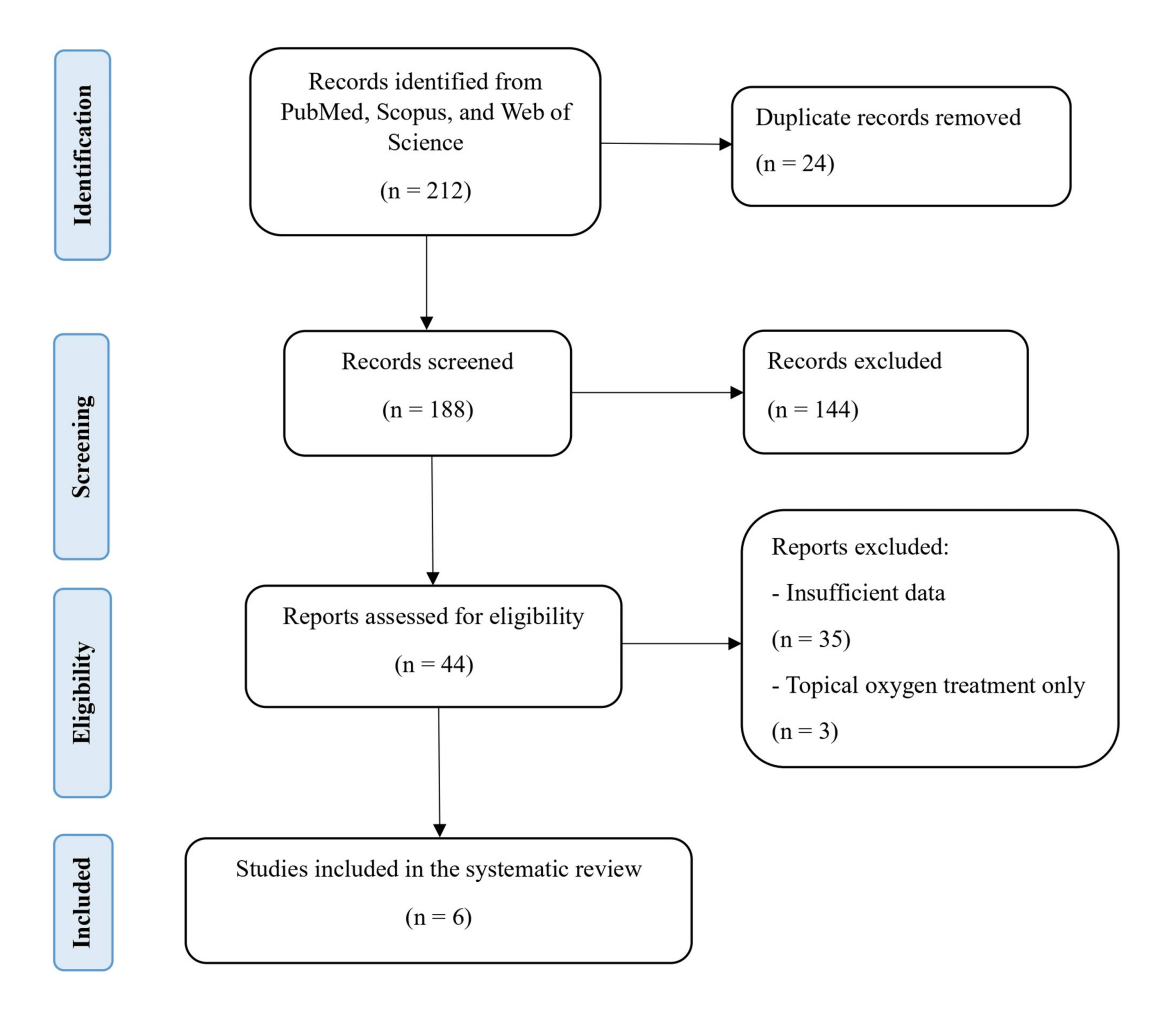
PRISMA flow diagram of the literature search and study selection for the systematic review PRISMA: Preferred Reporting Items for Systematic Reviews and Meta-Analyses

The initial search of PubMed, Scopus, and the Web of Science yielded 212 articles. After 24 duplicates were removed, 188 articles were screened. Subsequently, 144 articles were excluded due to unsuitable study designs, incomplete data, or not desired outcomes, such as amputation rates, ulcer healing, or wound size reduction. 44 reports were evaluated for eligibility, excluding 35 studies due to insufficient primary outcome data, incomplete statistical analyses, or unclear treatment protocols. Three more studies were eliminated, as they focused solely on topical oxygen treatment, not aligning with the objective of assessing systemic HBOT. Ultimately, six studies encompassing 391 patients met the inclusion criteria and were included in the systematic review [20,23-27]. The study selection process is illustrated in the PRISMA flow diagram in Figure 1, and Table 1 summarizes the key characteristics and findings of the included studies.

**Table 1:** Summary of selected studies on the primary clinical evidence of HBOT for the management of DFUs DFU: Diabetic foot ulcer; HBOT: Hyperbaric oxygen therapy; SC: Standard care; OR: Odds ratio; CI: Confidence interval

Study details	Objective	Sample size	Mean age (years)	Number of sessions and follow-up	Main results
Fedorko et al. [20]	Assess the efficacy of HBOT in reducing amputation rates and enhancing wound healing in chronic DFUs.	HBOT: 49 patients; Control: 54 patients; Total: 103 patients	HBOT: 61; Control: 62	30 sessions; 6-week follow-up	No significant reduction in amputation (51% vs. 48%; OR: 1.12; P = 0.771) or improvement in wound healing (20% vs. 22%; OR: 0.90; P = 0.823).
Perren et al. [23]	Investigate the effectiveness of HBOT combined with SC for ischemic DFUs.	HBOT: 13 patients; Control: 13 patients; Total: 26 patients	SC + HBOT group: ≤70 (7 patients) and ≥70 (6 patients); SC: ≤70 (7 patients) and ≥70 (6 patients)	40 sessions; 4-week follow-up	HBOT resulted in greater reduction in wound size (3.75 cm² vs. 1.05 cm²) and ulcer depth (0.89 cm vs. 0.19 cm). (P < 0.001).
Santema et al. [24]	Evaluate the benefits of HBOT for diabetes-related ischemic leg ulcers.	HBOT: 60 patients; Control: 60 patients; Total: 120 patients	HBOT: 67.6; Control: 70.6	40 sessions; 12-month follow-up	Improved limb preservation (13%; 95% CI: 2–28%) and amputation-free survival (OR: 10%).
Nik Hisamuddin et al. [25]	Evaluate the impact of HBOT on chronic DFU wound healing.	HBOT: 29 patients; Control: 29 patients; Total: 58 patients	HBOT: 54.4; Control: 57.9	30 sessions; 4-week follow-up	Wound size reduction: OR for at least 30% reduction was 44 times higher with HBOT (P < 0.001).
Salama et al. [26]	Investigate the role of HBOT in healing chronic nonischemic DFUs.	HBOT: 15 patients; Control: 15 patients; Total: 30 patients	HBOT: 55.1; Control: 57.7	20–40 sessions; 4–8-week follow-up	Complete healing achieved in 5 HBOT patients (33.3%) vs. 0 in the control group (P = 0.014).
Kumar et al. [27]	Evaluate HBOT as an adjunct to SC in DFU treatment.	HBOT: 28 patients; Control: 26 patients; Total: 54 patients	HBOT: 58.4; Control: 56.9	36 sessions; 6-week follow-up	Complete healing achieved in 78% of HBOT group (P = 0.001). All control patients required surgery.

The mean ages of participants in the HBOT (n = 194) and SC (n = 197) groups were 61.1 and 62.5 years, respectively. HBOT was delivered in 20-40 sessions, typically 90 minutes each session, with follow-up durations from two weeks to two months [20,23-27].

The analyzed studies suggest that HBOT yields better outcomes than SC in DFUs. In four of the six studies, HBOT reduced major amputation rates, with Santema et al. showing a 13% improvement in limb preservation [23-27]. HBOT led to higher rates of complete ulcer healing, exemplified by Salama et al.'s study where 33.3% of HBOT patients achieved full ulcer closure versus 0% in the SC group (P = 0.014) [26]. Significant improvements in ulcer size and depth reduction were observed in four of six studies, with Perren et al. reporting a more substantial decrease in ulcer size for patients with HBOT (3.75 cm² vs. 1.05 cm²) [23]. Amputation-free survival was higher among HBOT-treated individuals, with Santema et al. reporting a 26% absolute risk reduction in amputation [24]. Kumar et al. demonstrated faster wound closure times with HBOT, as 78% of treated patients achieved complete healing compared with the SC group (P = 0.001) [27]. While these findings underscore potential advantages of HBOT for DFUs, the diversity in study designs and patient cohorts necessitates additional large-scale, multicenter trials to draw definitive conclusions.

Multiple studies have demonstrated the efficacy of HBOT in the treatment of DFUs. Perren et al., Santema et al., Nik Hisamuddin et al., and Salama et al. reported significant reductions in ulcer area and depth following HBOT [23-26]. Although Fedorko et al. reported no significant difference in major amputation rates or long-term wound healing between the HBOT and SC groups, their results may have been affected by performance and detection bias [20]. Santema et al. found a 26% absolute risk reduction for amputation-free survival and a 10% improvement in limb preservation with HBOT [24]. Nik Hisamuddin et al. and Salama et al. highlighted the effectiveness of HBOT at reducing wound size and achieving complete ulcer healing, with Salama et al. noting 33.3% of the HBOT group achieving full closure versus none in the control group (P = 0.014) [25,26]. Kumar et al. further demonstrated the superiority of HBOT, showing complete ulcer healing in 78% of HBOT-treated patients compared to reliance on surgical interventions of those in the control group (P = 0.001) [27]. These findings suggest that HBOT is an effective adjunctive therapy for DFUs; however, variations in methodological rigor and potential biases necessitate further high-quality RCTs to confirm these results and optimize treatment protocols.

**Figure 2:**
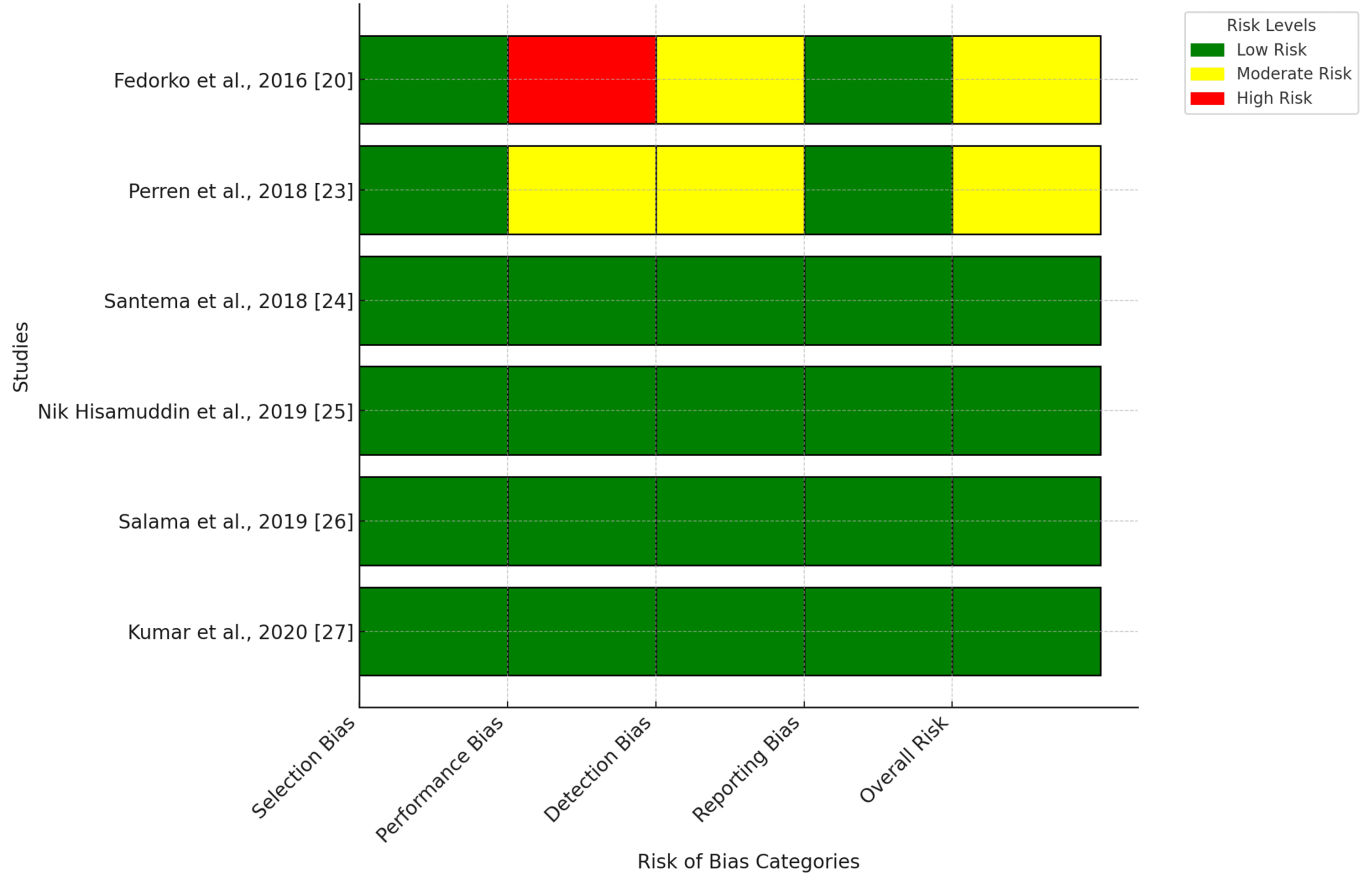
Risk of bias in individual studies of HBOT for the management of DFUs included in the systematic review DFU: Diabetic foot ulcer

This review evaluated RCTs using the RoB 2.0 tool, highlighting performance and detection bias as primary issues. Performance bias was notable in studies with insufficient blinding of participants and providers, which affected wound care practices and healing assessments (Figure 2). Fedorko et al. exhibited high performance bias, influencing treatment adherence and evaluations [20]. Detection bias was evident in studies in which outcome assessors were not blinded, leading to systematic differences in evaluating ulcer healing and amputation rates. Perren et al. showed a moderate detection bias, impacting wound measurement accuracy [23]. Most RCTs demonstrated low risks of selection and reporting bias owing to well-implemented randomization and allocation concealment. Attrition bias was observed in studies with incomplete follow-up data, which affected the long-term outcome evaluations. These biases could have led to over- or underestimation of HBOT effectiveness, emphasizing the need for well-blinded multicenter RCTs with standardized protocols to reduce bias and enhance reliability.

In observational studies evaluated using the ROBINS-I tool, the main issues identified were confounding, selection, and measurement biases. Confounding bias emerged when studies failed to adequately control for variables, such as comorbidities, ulcer severity, and initial patient characteristics, potentially skewing the estimated impact of HBOT. Selection bias was significant because patient allocation to HBOT or SC often relied on clinical judgment rather than randomization, compromising treatment group comparability. Measurement bias arose due to inconsistent assessment of wound healing outcomes, with variations in the follow-up periods and evaluation methods. Outcome reporting bias was evident in studies lacking predefined endpoints or inconsistent definitions of ulcer healing and amputation rates. These shortcomings limit the applicability of our findings, underscoring the need for observational studies with uniform outcome measures, consistent follow-up durations, and methodologies that incorporate propensity score matching or alternative confounding adjustment techniques.

We evaluated the outcomes of key studies. Four studies demonstrated notable ulcer healing improvement with HBOT and SC, whereas Fedorko et al. observed no significant change, which was attributed to increased performance and detection bias [20,24-27]. Perren et al. and Nik Hisamuddin et al. noted significant ulcer size and depth reduction with HBOT, with a cautionary note about the moderate risk of bias in the study by Perren et al. [23,25].

Studies by Santema et al., Salama et al., and Kumar et al. consistently found that HBOT decreased the incidence of major amputations compared to SC alone, a finding not replicated in the study by Fedorko et al., likely due to its higher risk of bias [20,24,26,27]. Salama et al. and Kumar et al. reported faster healing rates with HBOT, suggesting its efficacy as a supplementary therapy [26,27]. Despite the low risk of bias, caution is advised due to the moderate to high risk of bias in the studies by Fedorko et al. and Perren et al. [20,23]. Further RCTs with stringent blinding and methodology are critical for validation.

This review indicates that, compared with standard treatments, HBOT enhances ulcer healing, reduces ulcer size, and decreases amputation rates in patients with DFUs [24-27]. However, caution is necessary when interpreting results from studies with higher risks of bias, such as the study by Fedorko et al. [20]. More high-quality RCTs are needed to confirm the clinical efficacy of HBOT for managing DFU and to establish definitive treatment protocols.

Discussion

In this systematic review, we evaluated the use of HBOT for DFUs, indicating its potential as a complementary treatment. Most studies reported improved ulcer healing, reduced wound size, and lower amputation rates, though these results should be interpreted in the context of the methodological quality and biases of the studies included in the review [20,23-27].

HBOT is often the last resort to save limbs in cases of severe DFUs that are unresponsive to other treatments. Conflicting results regarding the effectiveness of HBOT for DFU treatment stem from several factors. Diverse research approaches, including RCTs and observational studies, have led to varied outcomes. Additionally, the lack of a uniform HBOT protocol with differences in pressure levels, session length, and total treatment cycles complicates direct comparisons between studies [19,24,28]. These elements collectively result in discrepancies, highlighting the need for large-scale multicenter studies with consistent methodologies to determine the efficacy of HBOT. In this review, we examined the existing literature on the use of HBOT for the treatment of DFUs to enhance the scientific discussion.

Conventional DFU management includes debridement methods (mechanical, surgical, autolytic, enzymatic, and larval), pressure relief strategies (specialized footwear, hosiery, sandals, insoles, and orthotic devices), dressings, and topical applications (hydrocolloids, hydrogels, foams, films, and silver-impregnated dressings) to promote wound healing [11,12,29]. Recent findings include growth factors, bioengineered skin substitutes, electrical stimulation, ultrasound therapy, and negative-pressure wound treatment [29]. However, variability in SC protocols across studies may affect HBOT's perceived advantages of HBOT. Differences in wound management, infection control, and supplementary treatment can affect the effectiveness of HBOT. SC in future clinical trials is essential to accurately determine HBOT's impact of HBOT and to enhance study comparability essential.

SC is the best-recognized treatment for a condition, based on clinical guidelines, evidence-based studies, and expert agreement. In DFUs, SC involves wound cleaning, managing infections, reducing pressure, and controlling blood sugar levels [12,29]. Key medications in DFU SC include broad-spectrum antibiotics such as amoxicillin-clavulanate, clindamycin, or piperacillin-tazobactam for infections. Blood-thinning drugs, such as aspirin or clopidogrel, enhance circulation, while medications that dilate blood vessels (e.g., cilostazol, pentoxifylline) boost blood flow in ulcers with poor circulation. Common topical treatments include silver sulfadiazine, iodine-based dressings, and platelet-derived growth factor (PDGF) (becaplermin) to promote healing. Proper blood sugar levels are vital for DFU management, with medications such as metformin, insulin, and sodium-glucose cotransporter-2 inhibitors for glucose control and ulcer prevention. In challenging cases, additional therapies, such as negative pressure wound therapy, HBOT, and bioengineered skin grafts, may improve healing. Establishing an SC ensures uniformity of treatment and serves as a benchmark for evaluating new therapies [12,29].

Several studies have suggested that HBOT may lower amputation rate in patients with DFUs [23-27]. These studies revealed that the SC group had higher frequencies of minor, major, and overall amputations, particularly major amputations, than the HBOT group. Santema et al. reported a decrease in major amputations in patients treated with HBOT versus SC [24]. A review by Stoekenbroek et al. determined that HBOT significantly lowered the major amputation rates in patients with ischemic ulcers [30]. However, some studies have observed increased minor amputations, suggesting a shift rather than an absolute decrease. This pattern might be attributed to enhanced limb preservation techniques, in which prompt minor amputations prevent more extensive limb loss.

Additionally, three RCTs suggested that HBOT improves DFU healing rates [20,23,25]. Other clinical studies have supported the efficacy of HBOT in treating DFUs [24,26,27]. Löndahl et al., Tiaka et al., and Kranke et al. found that 50% of patients in the HBOT group achieved complete ulcer healing compared to 29% in the conventional therapy group [16,31,32].

The accelerated healing of DFUs treated with HBOT may be attributed to the ability of HBOT to promote angiogenesis, enhance fibroblast proliferation, stimulate collagen production, and improve wound healing [33]. The angiogenic effects of HBO stem from the persistent elevation in oxygen tension after treatment. When oxygen levels reach or exceed 30-40 mmHg, ROS production in immune cells, such as neutrophils and macrophages, is enhanced, which is vital for eliminating bacteria. This increased ROS-mediated antimicrobial action boosts superoxide enzyme activity and improves the oxidative bursts that combat aerobic and anaerobic bacteria. Increased oxygen availability enhances immune cell functionality, bolstering the host's ability to fight infections [30].

HBOT enhances antibiotic efficacy by boosting tissue oxygenation, improving drug penetration into oxygen-deprived wound regions, and enhancing the localized antimicrobial action. Elevated oxygen levels can increase the absorption and dissemination of certain antibiotics, particularly those that are dependent on active transport mechanisms or oxygen-reliant bacterial elimination. Furthermore, enhanced oxygenation may amplify the bactericidal effects of specific antibiotics, including aminoglycosides, trimethoprim, nitrofurantoin, and sulfisoxazole, which demonstrates increased effectiveness in oxygen-rich settings [28,34].

Restoring the intermittent oxygen supply through swelling and poor blood-flow barriers helps maintain cellular structure and function, aiding the healing of poorly perfused tissues [28,34]. HBOT reduces platelet aggregation, improves microcirculation, and addresses metabolic issues, thereby enhancing oxygen delivery to deprived tissues via increased oxygen dissolution in the plasma [29].

Santema et al., Salama et al., and Kumar et al. demonstrated that HBOT significantly reduced major amputation rates and improved ulcer healing in patients with DFUs [24,26,27]. Salama et al. reported complete ulcer closure in 33.3% of patients in the HBOT group compared to none in the SC group (P = 0.014), while Santema et al. found a 13% improvement in amputation-free survival [24,26]. Perren et al. and Nik Hisamuddin et al. also noted significant decreases in ulcer size and depth with HBOT, suggesting its potential to enhance tissue oxygenation and stimulate angiogenesis [23,25].

Perren et al. and Santema et al. reported that HBOT benefits the long-term healing of ischemic DFUs more than that of nonischemic diabetic ulcers [23,24]. Patients with ischemic DFUs who underwent HBOT had a significantly lower major amputation rate [24,27]. However, adjuvant HBOT increased major amputation rates without aiding in the healing of ischemic DFUs [19,20]. These conflicting results highlight the need for further research on this patient group.

In the included studies, "major amputation" typically referred to procedures above the ankle, including below-knee and above-knee amputations. "Minor amputations" were generally those at or below the ankle level, such as toe or partial foot amputations [24,26,27,30]. These definitions vary among studies, potentially affecting the outcome comparisons. Some studies differentiated between transtibial and transfemoral procedures, whereas others grouped all above-ankle amputations [20,23]. This classification inconsistency should be considered when evaluating amputation-related results.

The evaluation of outcomes, such as wound healing rates, reduction in ulcer dimensions, and improvements in wound depth, employed diverse techniques and timelines across studies [24,25,28]. Studies have used digital planimetry, wound tracing, photographic evaluation, and clinical assessment [23,29]. The follow-up duration varied from weeks to months, complicating direct comparisons of HBOT effectiveness [20,24,27]. These methodological variations may account for the observed heterogeneity in the results, and should be considered when evaluating the efficacy of HBOT in managing DFUs.

The risk of bias varied among the studies included in the review. Many studies exhibited a low risk of selection, attrition, and reporting bias, indicating reliable randomization, adequate participant retention, and transparent reporting. For instance, the studies by Santema et al., Nik Hisamuddin et al., Salama et al., and Kumar et al. showed low risk levels across most domains, enhancing the credibility of the findings and linking HBOT to improved ulcer healing and reduced amputation risk [24-27].

A significant constraint impacting evidence robustness is performance bias, primarily because of inadequate blinding in multiple studies. Fedorko et al. demonstrated high performance bias, potentially influencing wound healing evaluations [20]. Perren et al. exhibited a moderate detection bias risk, raising questions about outcome measurement objectivity [23]. Furthermore, selection bias was evident in certain studies owing to limited sample sizes and nonrandomized designs. While Salama et al. and Kumar et al. reported notable improvements in ulcer healing with HBOT, their small sample sizes may restrict the applicability of their results [26,27]. Selection bias might have influenced studies in which patients with more severe ulcers were preferentially assigned to HBOT, potentially skewing the outcomes.

The diversity in HBOT protocols, follow-up periods, and outcome measures further complicated the interpretation of the results. Although Fedorko et al. reported no significant reduction in amputation rates or improved long-term healing, this might stem from methodological weaknesses and design differences [20]. Conversely, the studies by Santema et al. and Kumar et al., which had superior methodological quality, reported significant benefits of HBOT, including lower major amputation rates and higher complete ulcer healing rates [24,27].

This systematic review indicated that HBOT may enhance ulcer healing, decrease wound dimensions, and reduce amputation frequency in patients with DFU. However, the small number of studies and modest sample size could impact the strength of these conclusions. The heterogeneity in patient populations, ulcer severity, and comorbid conditions across studies may have affected the broader applicability of the outcomes. Variations in HBOT protocols, follow-up durations, and measurement methods further challenge the interpretation of findings. To strengthen reliability, future research should focus on large-scale high-quality RCTs with uniform methodologies and clearly defined patient selection criteria. Further studies are also needed to assess the proportion of patients with fully healed DFUs randomized to the HBOT and control groups.

## Conclusions

The findings of this systematic review indicated that HBOT for DFUs results in lower amputation rates and significant improvements in lesion size and wound healing speed compared with conventional methods. Although there are indications of its positive effects on wound healing, it remains unclear which patients most benefit from HBOT. To justify its widespread adoption in standard practice, its efficacy must be validated through comprehensive RCTs with strict methodological guidelines and consistent outcome measures.

To confirm these results across clinical settings, patient groups, and healthcare environments, large-scale, multicenter studies are necessary. These investigations should minimize bias, create uniform treatment protocols, and determine which patient subsets are most likely to respond to HBOT. Most studies demonstrated a generally low risk of bias, although some studies showed moderate to high bias, which requires careful consideration. Evaluating the cost-effectiveness of HBOT and its effects on patient quality of life is crucial for informed clinical decisions. Given resource-intensive nature of HBOT, assessing its economic viability and clinical advantages will help determine its long-term practicality in regular medical practice. Future studies should prioritize rigorous blinding procedures, consistent outcome assessments, extended follow-up periods, and multicenter involvement to ensure more dependable and applicable results.
